# Soil evaporation and organic matter turnover in the Sub-Taiga and Forest-Steppe of southwest Siberia

**DOI:** 10.1038/s41598-018-28977-8

**Published:** 2018-07-19

**Authors:** Zachary E. Kayler, Félix Brédoire, Helene McMillan, Pavel A. Barsukov, Olga Rusalimova, Polina Nikitich, Mark R. Bakker, Bernd Zeller, Sébastien Fontaine, Delphine Derrien

**Affiliations:** 1 Institute for Landscape Biogeochemistry, Leibniz Center for Agricultural Landscape Research, Müncheberg, Germany; 2 Department of Soil and Water Systems, University of Idaho, 875 Perimeter Drive, Moscow, ID United States of America; 3 Center for Accelerator Mass Spectrometry (CAMS), Lawrence Livermore National Laboratory, 7000 East Ave, 94550 CA Livermore, United States of America; 4 INRA, UR 1138 BEF, 54280 Champenoux, France; 5 INRA, UMR 1391 ISPA, 33140 Villenave d’Ornon, France; 6 Bordeaux Sciences Agro, UMR 1391 ISPA, 33140 Villenave d’Ornon, France; 7 Federal Institute for Geosciences and Natural Resources (BGR), Stillweg 2, 30655 Hannover, Germany; 8 Institute of Soil Science and Agrochemistry, Novosibirsk, Russia; 9 INRA, UREP, 5 Chemin de Beaulieu, 63000 Clermont Ferrand, France

## Abstract

Southwest Siberia encompasses the forest-steppe and sub-taiga climatic zones and has historically been utilized for agriculture. Coinciding with predicted changes in climate for the region is the pressure of agricultural development; however, a characterization of the soil water and carbon dynamics is lacking. We assessed current soil water properties and soil organic carbon turnover in forests and grasslands for two sites that span the forest steppe and sub-taiga bioclimatic zones. Soil evaporation was 0.62 ± 0.17 mm d^−1^ (mean ± standard error) in grasslands and 0.45 ± 0.08 mm d^−1^ in the forests of the forest-steppe site. Evaporation at the sub-taiga site was 1.80 ± 1.70 mm d^−1^ in grasslands and 0.96 ± 0.05 mm d^−1^ in forest plots. Evaporation was significantly greater at the sub-taiga site than the forest-steppe site. The density of fine roots explained the soil water isotopic patterns between vegetation types and sites. We found soil organic matter turnover to be three times faster in the sub-taiga site than in the forest-steppe site. Our results show that while climate factors, in particular snow levels, between the two sites are drivers for water and carbon cycles, site level hydrology, soil characteristics, and vegetation directly interact to influence the water and carbon dynamics.

## Introduction

**Figure 1:**
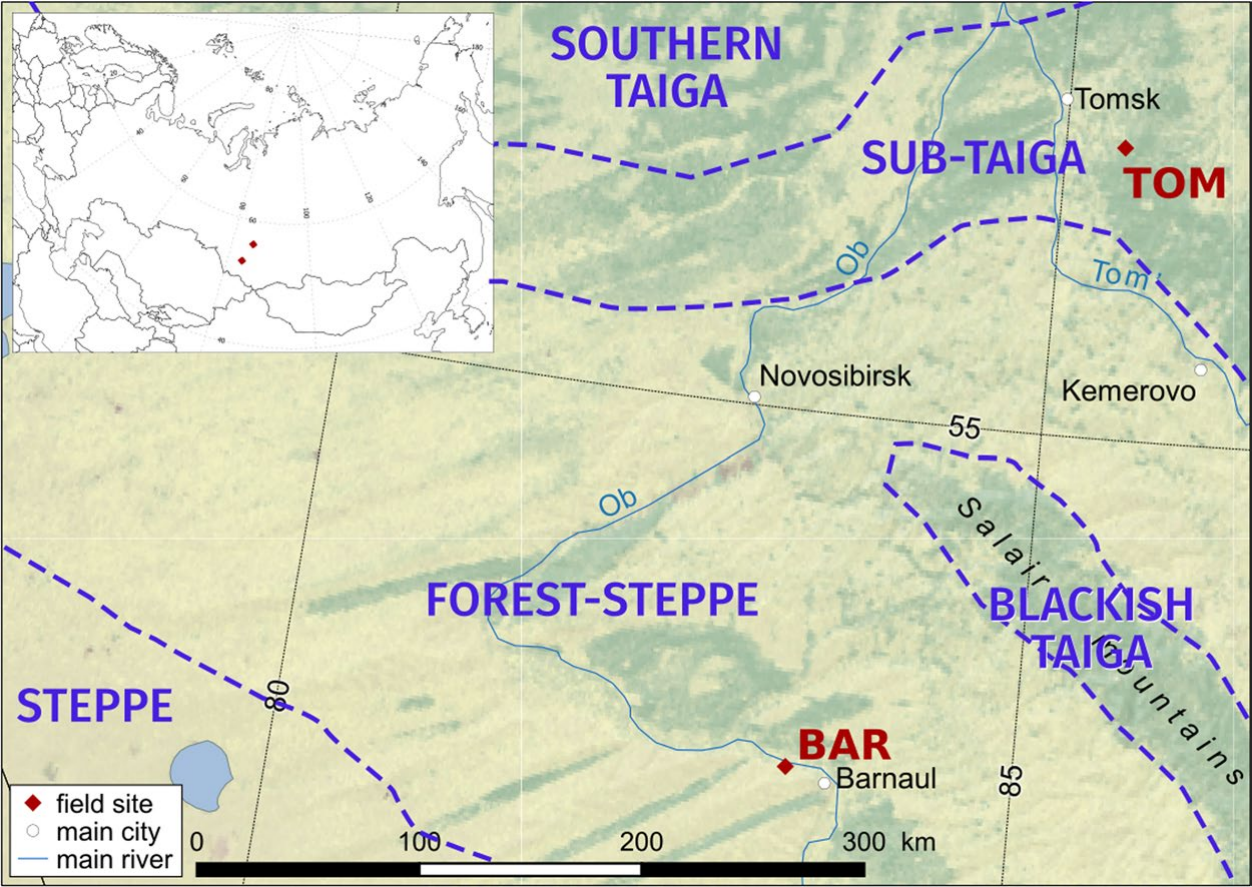
Sites selected in southern Siberia and the corresponding bioclimatic zones. Inset displays the sites within the northern Eurasian region. The map was created with QGIS version 2.4.0-1 (www.qgis.org) software.

The southwest region of Siberia encompasses both steppe and southern-taiga bioclimatic zones that contain grasslands and aspen-birch forests ecosystems growing on fertile soils and is currently used for agriculture and forest production. The southwest region (Fig. 1) is expected to experience several shifts in water and energy levels due to climate change^1^ and ecosystems have already responded as seen, for example, by the tree-line shift toward mountains ridges^2^. Along with changes in climate, expected changes in land-use are foreseen as the agricultural season in the region is potentially extended^3,4^. The region also contains nutrient rich soils, such as Chernozems, increasing the region’s likelihood for further agricultural conversion^5^. Many of these outcomes rely on the availability of water and nutrients released from soil organic matter to coincide with the lengthening of the season, shifts in phenology, or temperature increases, highlighting the need for field data of water and organic matter dynamics in this region^6^.

Changes in the precipitation regime are expected for the region in both seasonal inputs (snow vs. rain) and intensity (shift in distribution over the year). Already, the number of days of heavy rainfall and very heavy rainfall has increased and a shorter snow cover period in some Arctic areas has also been observed^7–9^. For SW Siberia, an increase in snow depth is expected^9,10^. Snow cover in this region can be an important soil insulator, when snow pack is sufficient, by preventing soil freezing and allowing for biological processes, such as nutrient mineralization, to occur during the winter period^11^. Studies predict that precipitation changes and temperature increases can lead to a decrease in crop productivity^12^ while still other research predicts a two-fold increase^5^. Much of the uncertainty lies in the degree to which soil moisture in the south will be sufficient to grow new crops and the sustainability of current soil conditions (i.e., fertility)^5,13^.

Interspersed within the grassland and agricultural landscapes of SW Siberia are forests of Aspen (*Populus tremula* L.) and Birch (*Betula pendula* Roth.)^14^. The predicted shifts in precipitation could lead to a greater evaporation deficit, restricting tree growth^15^, highlighting the importance of soil moisture for forests in the southwest as well. In the forest-steppe region of SW Siberia, aspen growth is largely constrained by soil moisture, especially current year growth is highly influenced by the previous year’s soil moisture conditions, indicating snow levels as an important factor^16^. While further north, in the sub-taiga, aspen growth is heavily reliant upon summer air and soil temperatures^16^. Field research has been performed in Siberian boreal forests^17,18^; however, information regarding water loss through soil evaporation at the site scale is lacking for forests in the southwest region.

Plant available nutrients stored in soil are highly dependent on soil organic matter turnover, as suggested by the phosphorus status in sites across SW Siberia^19^. Furthermore, soil organic matter and carbon is known to improve soil water retention within the soil profile^20^. Given the often-slow turnover dynamics of soil organic matter^21^, especially in colder environments^11^, quantifying soil organic matter turnover can be a challenge. Characterizing the soil profile using the carbon stable isotopic values^22–24^ of soil organic matter is an established proxy that has been tied to soil physical properties, climate, and biota across a range of soil types^24^. Thus, information from the soil profile may provide an integrative assessment of organic matter dynamics and a means to investigate how these soil carbon dynamics are related to the water status at a site.

Roots play a crucial role across spatial scales from maintaining plant water status to contributing to ecosystem carbon and nutrient cycling^25,26^. The impact of roots on ecosystem water status extends beyond ensuring connectivity between plant and soil and includes the redistribution of soil water via hydraulic lift^27^, the maintenance of proximal sources of soil water^28^, and controls over plant response to water stress^29^. Spatial patterns in fine root distributions of grasslands and forests in sub-taiga and forest-steppe bioclimates have been found to be primarily driven by climate and vegetation type^30^. In the same study, differences in specific root length and vertical distribution between grassland and forest species were also reported. However, how the vertical distributions of fine roots or differences in vegetation type are related to available soil moisture within the soil profile is unknown and can potentially reveal patterns in plant accessible water pools belowground.

The current relationship between soil water and organic matter may give indication of future agricultural constraints. However, due to the vast size of southwest Siberia and its remoteness, there are logistical difficulties in bringing these dynamics to light. We took advantage of the soil profile to infer the status and rate processes of soil water and carbon using stable isotopes at two sites that capture the gradient in soil and climate conditions across the region. Stable isotopes integrate processes over multiple time scales. In the case of soil carbon, δ^13^C is a proven proxy of organic matter turnover that occurs over decades. Soil water δ^18^O, on the other hand, records the evaporation and precipitation dynamics over the season, not just the time period that the soil is sampled. They also indicate when different sources are important, such as groundwater, which can be a general characteristic of the ecosystem.

The sites we investigated encompass bioclimactic zones of forest-steppe to the south (Barnaul) and to the north, the sub-taiga (Tomsk). Within these sites we quantified soil evaporation and organic matter turnover for both forest and grassland vegetation types. The soil carbon content data and isotopic composition over the depth profile were combined into a linear model^31^ to assess the carbon turnover rate. Water was extracted from the same soil sample. Its isotope signatures provided information regarding isotopic source (i.e., precipitation, groundwater, etc.), processes such as evaporation^32,33^, and even age^34^.

Our overarching goal was to provide a first view of how soil water and carbon might be coupled within this region. We expected both climate (temperature and precipitation) and soil properties to constrain soil water evaporation and organic matter turnover. We hypothesized:

i. Greater soil evaporation rates associated with the relative greater temperatures at the southern forest-steppe Barnaul site compared to the sub-taiga Tomsk site.
ii. Faster rates of organic matter turnover at the southern forest-steppe site due to a longer growing season characterized by higher temperatures relative to the sub-taiga site.

Furthermore, we expected differences in vegetation to impact the water dynamics evidenced by the root distribution. We placed our results within the context of the growing pressure of agriculture in the region and the sustainability of the current soil health.

## Methods

### Site description

We selected two sites that represent the endpoints of a snow gradient across southwest Siberia (Table 1). The southern site is located near the city of Barnaul in the forest-steppe zone. The mean annual temperature is 2.7 °C (−14.1 °C in winter and 18.3 °C in summer) and the snow season (where snow depth >1 cm) lasts on average 157 days and the snow level is on average 49 cm deep at peak snow season. The northern site is located near the city of Tomsk in the sub-taiga zone. The mean annual temperature is 0.9 °C (−15.6 °C in winter and 16.7 °C in summer) and the snow season lasts on average 178 days and the snow level is on average 71 cm deep at peak snow season. The water table fluctuates during time periods at the Tomsk site, in which soil goes through periodic saturation. Consequently, clays are washed from the topsoil and accumulate in the deeper layers, and carbonates have disappeared from the first meter of the soil profile. There is no indication of tillage in the soil profile of Barnaul and Tomsk grasslands and management consists of occasional mowing, thus, both sites have been under current vegetation management conditions for approximately the last 100 yrs (P. Barsukov *personal communication*).

**Table 1:** Characteristics of sites located in the forest-steppe and sub-taiga regions of southwest Siberia.

Site	Barnaul (BAR)	Tomsk (TOM)
Bioclimatic Zone	Forest-steppe	Sub-taiga
Latitude N/Longitude E	53.41/83.47	56.3/85.43
Elevation (m)	221	232
Forest Soil	Haplic Phaeozem	Albic Luvisol
Grassland Soil	Calcic Chernozem	Albic Luvisol
Mean Annual Precipitation (mm)^†^	432	567
Mean Annual Temperature (°C)^†^	2.7	0.9
Forest Litterfall (g m^−2^)^‡,*^	364.0 ± 41.7	216.4 ± 33.6
Aboveground Grassland Biomass (g m^−2^)^*^	213.9 ± 4.7	299.0 ± 60.8

^†^Average over the period 1981–2010 from the closest weather station.

^‡^Collected between July and September of 2013.

^*^Biomass estimates from Brédoire^30^.

### Soil Samples

We sampled the sites at two time periods, once in June and July 2013; however, we were not able to detect a significant difference between the soil water isotopic composition between the two collections (spring mean δ^18^O = −15.7‰ vs. VSMOW, summer mean = −15.9‰, t = 0.296, df = 86.8, p = 0.77), thus we combined the data for analyses. At each site and each date, we sampled three different soil pits (i.e., 3 replicates) for each vegetation type (forest and grassland). Within each soil pit we collected soil material at six depths (3, 5, 15, 30, 60 and 100 cm). The soil was collected in 12 ml glass flasks, cooled in the field, then kept frozen until water extraction in the laboratory.

### Water extraction

The cryogenic extraction is based on the methods of Koeniger *et al*.^35^. We transferred a subsample of soil to a 5 ml glass vial with a cap equipped with a rubber butyl septa. The vial was briefly immersed in liquid nitrogen then connected via a stainless-steel tube that punctured the septa of both the sample and collection (12 ml) vials. The two vials connected by the stainless-steel tubing were placed under vacuum (−25 mTorr) for 5 minutes while the contents were still frozen. The soil sample vial was then inserted into an aluminum block heated to 110 °C. The collection vial was placed in a dewar of liquid nitrogen. The cryogenic extraction occurred over 45 min. Not all samples resulted in significant yields, resulting in different samples sizes for each depth (total *n* = 100, min = 1 and max = 6), but the average *n* across all plots was at 3 cm = 2, 5 cm = 3, 15 cm = 5, 30 cm = 5, 60 cm = 5, and 100 cm = 5. We provide a table of the sample size number in the Supplementary (S1). We had a consistent transfer of water (>98% by mass) and for each sample we calculated the gravimetric water content (%) on a dry weight basis. Based on water with known isotopic composition extracted from organic matter free sand, silt, loam, and clay mixtures the precision of the extraction was 0.27‰ for δ^18^O, and 1.20‰ for δ^2^H and a corresponding accuracy of 0.45‰ and 2.51‰.

### Isotope analysis

Stable oxygen (δ^18^O) and hydrogen (δ^2^H) isotope ratios of soil water were measured via cavity ring down spectrometry (CRDS). Water samples were vaporized at 110 °C (Isotopic H_2_O A0211) analyzed with a Picarro L2130-I Isotopic H_2_O analyzer (San Jose, CA, USA). Repetitive measurements of laboratory standards with the Picarro yielded a measurement precision of <0.1‰ for δ^18^O and <1.5‰ for δD. After water extraction, the soil samples were weighed into tin capsules and combusted in an elemental analyzer (Flash HT, Thermo Scientific, Bremen, Germany). The carbon stable isotope ratios (δ^13^C) of soil were measured with a Thermo-Scientific, Delta V Advantage isotope ratio mass spectrometer (Bremen, Germany).

Stable isotope ratios are reported in delta notation (in ‰ units) after equation (1), where the ratio *R* of the heavy isotope to lighter isotope in a sample is referenced to an international standard^36^:

1 $$\delta =(\frac{{R}_{sample}}{{R}_{standard}}-1)\times 1000$$

Isotope values are reported relative to Vienna Pee Dee Belemnite (VPDB) for carbon and to Vienna Standard Mean Ocean Water (VSMOW) for oxygen and hydrogen. Isotopic calibration for the EA-IRMS measurements was to IAEA-CH-6 (sucrose) and USGS40 (L-glutamic acid). Analysis of internal laboratory standards ensured that the estimates of the isotopic values were precise to within <0.1‰ for δ^13^C. Total organic carbon concentrations (in wt%) of soil samples were also obtained by EA-IRMS measurements.

### Data analyses

Rainwater was collected over the following year (n = 42, 2014) to develop a local meteoric water line (LMWL). The LMWL is primarily driven by larger climate controls and is not expected to deviate significantly inter-annually. If there were significant differences in soil evaporation we would expect to see deviations in the soil water δ^18^O, δ^2^H that are site dependent. We used the model of Zimmerman^32^ and Barnes^33^ to estimate soil evaporation based on the soil water profile δ^18^O values. We implemented measured δ^18^O values and literature values as input into the model; these are discussed below. The model was then optimized to derive evaporation estimates. The model is a steady-state model for a saturated water column:

2 $${\delta }^{18}{O}_{soil}=({\delta }^{18}{O}_{surface}-{\delta }^{18}{O}_{input})\times exp(\frac{-Z}{{Z}^{\ast }})+{\delta }^{18}{O}_{input}$$

where δ^18^O_soil_ is the soil water isotopic signal, δ^18^O_surface_ is the surface isotopic signal, δ^18^O_input_ is the original water source isotopic value, Z is the soil depth, and Z^*^ is the decay length (see eqn. 4). We assumed steady-state within the soil profile and used the soil water value measured at the deepest depth for δ^18^O_input_ since the intersect of the local enrichment line and meteoric water line can lead to biased results^37^. We determined δ^18^O_surface_ by eqn. 3 described in Chamberlain^38^.

3 $${\delta }^{18}{O}_{surface}={\varepsilon }^{+}\times [(1-h)\times {\varepsilon }_{k}\times {\delta }^{18}{O}_{input}+h\times {\delta }^{18}{O}_{vapor}]$$

where ε^+^ is the equilibrium fractionation factor in water, *h* is the relative humidity, ε_k_ is the kinetic isotope fractionation factor – a function of *h*^39^, and δ^18^O_vapor_ is the isotopic composition of water vapor at the surface. Evaporation is given in the decay length (Z^*^) described by eqn. 4:

4 $${Z}^{\ast }=\frac{{D}^{\ast }}{E}$$

Here, E is soil evaporation (m^3^ water m^−2^ s^−1^) and D^*^ is the effective diffusivity (m^2^ s^−1^) of δ^18^O in soil water and is given by the product of soil porosity, tortuosity and the diffusivity of δ^18^O in water. We used a value of 0.5 for porosity and tortuosity^38^, and a δ^18^O diffusion value of 2.66 × 10^−9^ m^2^ s^−1^ ^40^. We fit the data to the model using a non-linear regression in R^41^ to determine evaporation (E), and δ^18^O_vapor_. We tested for significant evaporation differences between vegetation and site using a z-test.

To test for the root distribution relationship to the soil water δ^18^O isotopic signature, we used a linear mixed-effects model (δ^18^O ~ site-vegetation factor + fine root mass density + fine root mass density:depth + (1|depth)). The interaction term was included to account for the decreasing patterns of root density with depth. The fine root mass density (FRMD g cm^−3^) was previously reported^30^ and the data used here are provided in the Supplementary material (S2). Significant differences between sites and vegetation were tested at the α = 0.05 level and were determined via a Tukey post-hoc test using the R package *multcomp 1.4-1*^42^. We used an ANOVA to test for differences (α = 0.05) in soil moisture content between sites, vegetation, and depth.

We also analyzed the δ^13^C isotopic signal from the soil after water extraction. We tested for differences between site and vegetation using an ANOVA followed by a Tukey test (α = 0.05 level). The δ^13^C patterns along the profile provide an opportunity for us to consider the degree of organic matter turnover. Microbial transformation (i.e., decomposition), for example, is one of several mechanism that will change the relative ratios of ^13^C/^12^C^36^. Based on the model of soil carbon content and the isotopic composition of soil carbon in the depth profile a linear model can be fit, the slope of this fit is often related to climate and soil type^31^. We used the concentration calculation from eqn. 5, to estimate a fraction of the surface organic matter remaining, implemented as *F*_*z*_ in eqn. 6.

5 $$C(z)={C}_{s}-(\frac{{J}_{s}}{D}\times \bar{z})\times (1-{e}^{\frac{-z}{\bar{z}}})$$

*C* (g C/g soil) is carbon concentration at depth *z*, *D* is the biodiffusion coefficient for SOC, C_s_ is the soil carbon concentration at the surface, *J*_*s*_ is the flux at the surface (*C* cm^−2^ s^−1^), $\bar{z}$ is the e-folding depth (production depth at which production is equivalent to the production at the surface relative to *e*). We used our data to fit the terms *C*_*s*_, *Js/D*, and $\bar{z}$ in a non-linear regression. From this analysis, we could calculate the proportion of soil carbon at depth z relative to the surface concentration (*C*_*s*_). We then used a model^43,44^, to characterize the relationship between the proportion of *C/C*_*s*_ at depth z to the isotopic composition of soil organic matter at depth *z*:

6 $${\delta }^{13}{C}_{z}={\delta }_{i}+\varepsilon \times \,\mathrm{log}({F}_{z})$$

In this model, the soil organic carbon isotopic composition (δ^13^C) at depth *z* is described as a function of the initial organic matter found at the surface (i.e., *C/C*_*s*_ = 1) and its isotopic value (δ_*i*_), the soil carbon profile turnover parameter (ε), and log of the fraction of surface soil organic matter remaining at depth *z* (*F*_*z*_, determined from eqn. 5). We fit our data to estimate δ_*i*_ and ε.

The datasets generated during and analyzed during the current study are available from the corresponding author on reasonable request.

## Results

### Soil water δ^18^O and δ^2^H profiles

**Figure 2:**
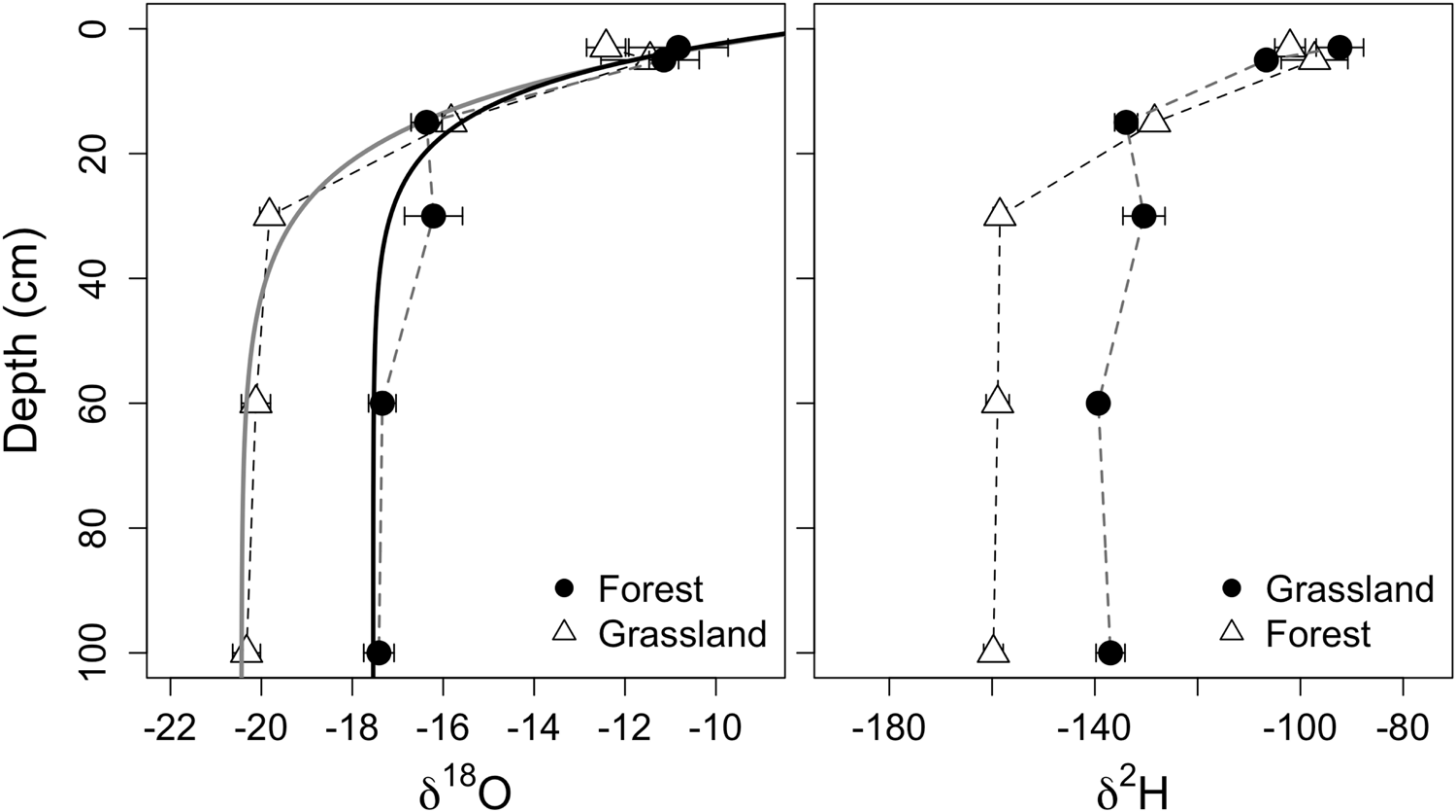
Soil water δ^18^O (left) and δ^2^H (right) isotopic profiles from the grassland and forest vegetation types at the forest-steppe site Barnaul (mean ± s.e.). The solid lines are the soil water δ^18^O isotopic profile model results used to estimate soil evaporation.

**Figure 3:**
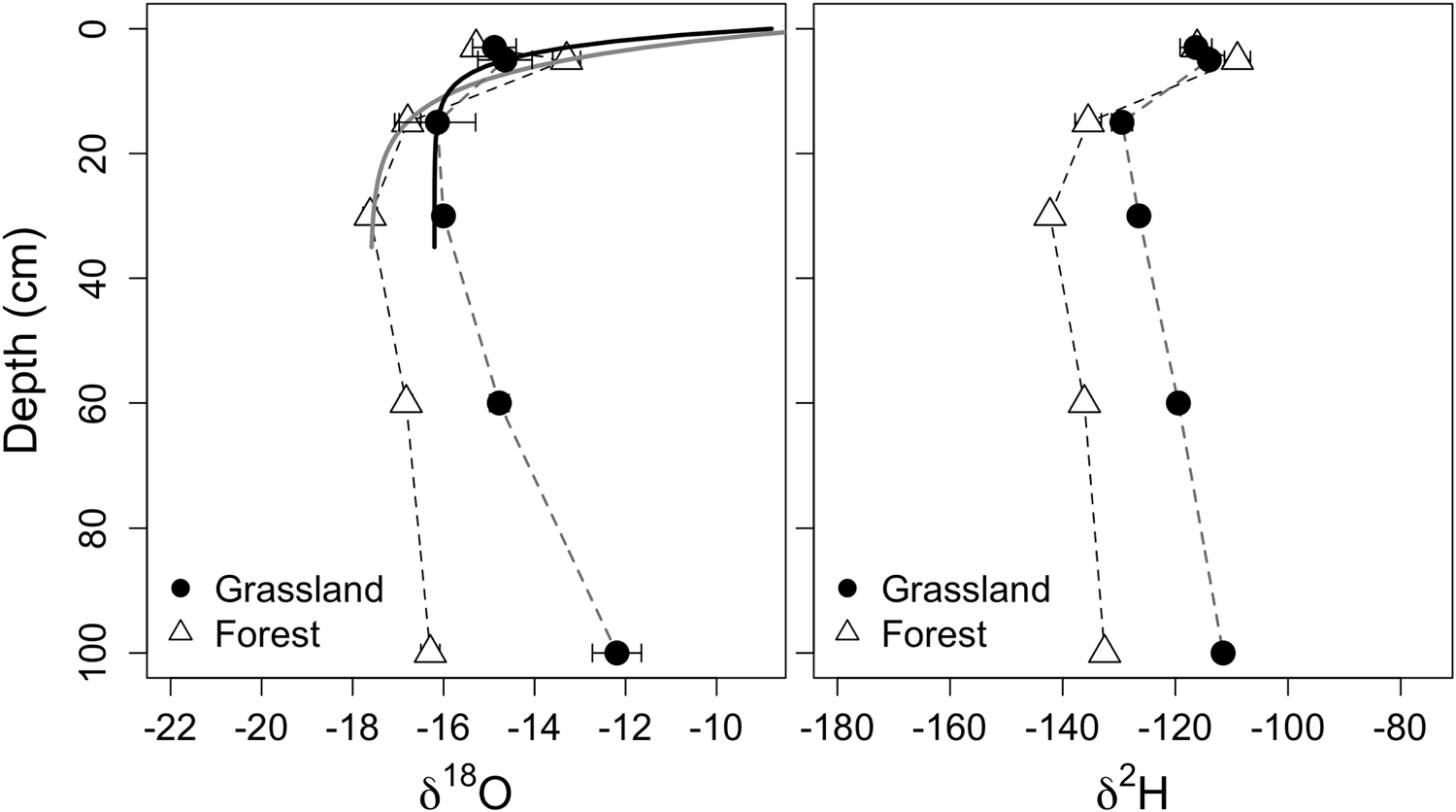
Soil water δ^18^O (left) and δ^2^H (right) isotopic profiles from the grassland and forest vegetation types at the sub-taiga site Tomsk (mean ± s.e.). The solid lines are the soil water δ^18^O isotopic profile model results used to estimate soil evaporation.

Distinct patterns in the soil water isotopic profile existed at both sites creating unique profiles (Figs 2 and 3). The range in δ^18^O spanned −9.8 to −19.3‰ within the Barnaul grassland (n = 22) and −9.9 to −22.5‰ within the forest (n = 28). The range in δ^2^H spanned −85.8 to −148.5‰ within the grassland (n = 15) and −88.1 to −175.7‰ within the forest (n = 22). The water isotopic values were most enriched at 3 to 5 cm depth, demarking the evaporative front in the soil, and then became increasingly depleted with depth.

At the Tomsk site, the range in δ^18^O spanned between −12.7 to −18.4‰ in grassland and between −10.6 to −17.3‰ in forest. The range in δ^2^H spanned −105.0 to −146.4‰ within the grassland and −107.5 to −137.3‰ within the forest. The water isotopic values were enriched in the heavy isotopes at 3 to 5 cm depth, demarking the evaporative front, followed by increasingly negative values until 30 cm. At depths deeper than 30 cm, the values show a pattern of enrichment again (i.e., resulting in a C – shaped profile).

**Figure 4:**
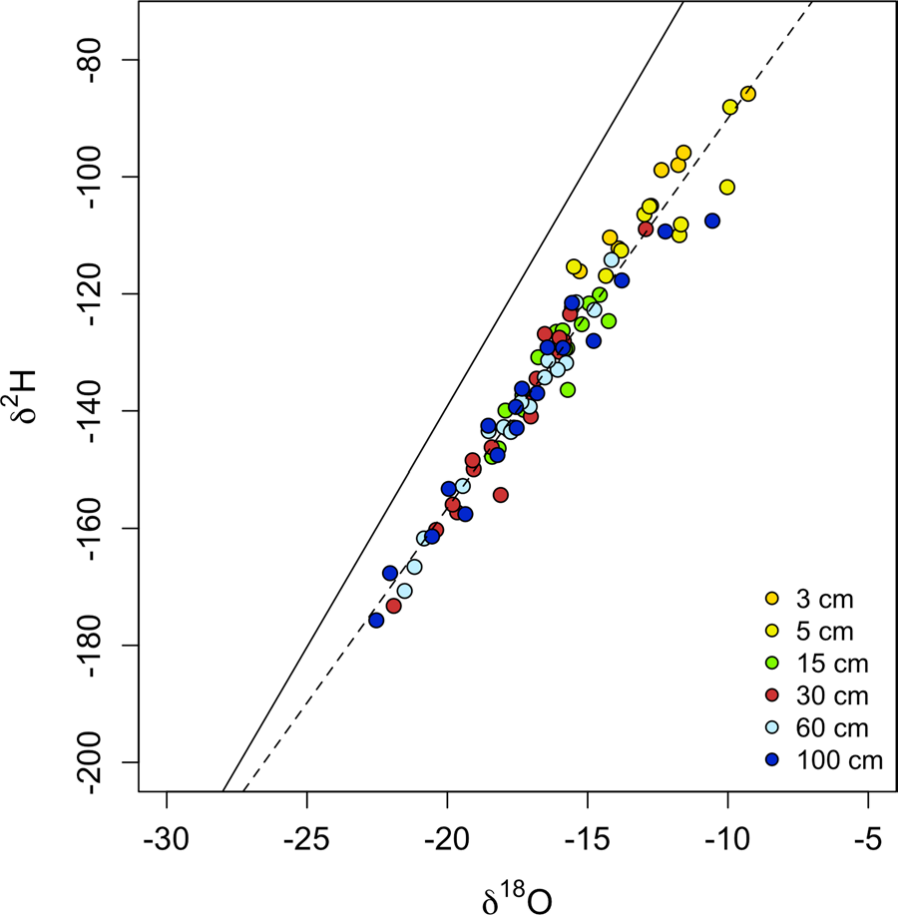
Soil water isotope values from both sites and vegetation types combined. The local meteoric water line (LMWL) derived from local precipitation is depicted by a solid line. Extracted soil water isotopic values from different depths are depicted by the different colored symbols. The local enrichment line (LEL) is plotted through the soil water data and is indicated by the dotted line.

The local meteoric water line (LMWL) determined by the local precipitation (δ^2^H ~ 8.2 × δ^18^O + 25.2) is depicted in Fig. 4, the soil water from both sites plot off from the LMWL on a local enrichment line (δ^2^H ~ 6.6 × δ^18^O + −23.4).

### Evaporation estimates

We were able to use the δ^18^O profile to estimate evaporation using the Zimmerman model^32^ (Table 2., Figs 2 and 3). The evaporation rate at the soil surface in the grassland of Barnaul was 0.62 ± 0.17 mm d^−1^ (mean ± standard error) and 0.45 ± 0.08 mm d^−1^ in the forest. These rates were not significantly different. We could not use the full soil profile at the Tomsk site to estimate soil evaporation due to the increasingly enriched water in the lower profile. We thus limited the evaporation analysis to the data within 30 cm depth, reducing sample size, which resulted in poor fits with large uncertainty. The evaporation estimate of surface evaporation in grasslands at the Tomsk site was 1.80 ± 1.70 and 0.96 ± 0.05 mm day^−1^ in forest plots. Because of the large uncertainty in the Tomsk estimates, we could not test for differences between vegetation types. Evaporation at the Tomsk forest, based on the 30 cm profile, was greater (0.51 ± 0.01 mm day^−1^) than evaporation at the Barnaul forest plots (Z-test, n = 2, p < 0.01).

**Table 2:** Model parameters used to fit water δ^18^O profile and derive evaporation estimates (eqs 2–4).

Parameter	Barnaul		Tomsk	
	Forest	Grassland	Grassland	Forest
δ ^18^O_input_ (‰)^†^	−20.4	−17.5	−17.6	−16.2
δ^18^O_vapor_ (‰)^‡^	−0.5	−2.3	−2.1	−4.6
E (m^3^H_2_O m^−2^ s^−1^) (standard error)	5.24 × 10^−9*^ (1.03 × 10^−9^)	7.21 × 10^−9*^ (1.94 × 10^−9^)	1.11 × 10^−8*^ (5.85 × 10^−10^)	8.83 × 10^−9*^ (1.41 × 10^−9^)

^†^Values entered in the model.

^‡^Modeled coefficients were not significant.

^*^Significant at p < 0.05 level.

### Statistical model

The linear model explained 58% of soil δ^18^O variance based on the mixed model latent scale (R2 marginal) and 66% of the overall (i.e., both fixed and random factors, R2 conditional) variation^45^ in soil water δ^18^O data, furthermore, the fine root mass density was highly significant (Table 3).

**Table 3:** Coefficients, variance, and significance of linear mixed-model to explain measured soil δ^18^O.

	Coefficient	Std. Error	DF	t-value	p-value
Barnaul Forest (intercept)	−17.11	0.85	78	−20.2	0
FRMD	3.50	0.73	78	4.8	0
Barnaul Grassland	0.81	0.55	78	1.50	NS
Tomsk Forest	0.22	0.67	78	0.33	NS
Tomsk Grassland	1.61	0.73	78	2.21	0.03
FRMD:Depth	−0.14	0.025	78	−5.74	0

n = 89.

The post-hoc Tukey tests of the model revealed significant differences between sites and vegetation types (p < 0.05). There was a significant difference of +1.4‰ (se = 0.56) between the grassland and forest soil water δ^18^O at the Tomsk sub-taiga site. Across sites, the Barnaul forest-steppe grassland soil was also +1.4‰ (se = 0.57) enriched relative to the Tomsk forest soils.

### Soil water content

**Figure 5:**
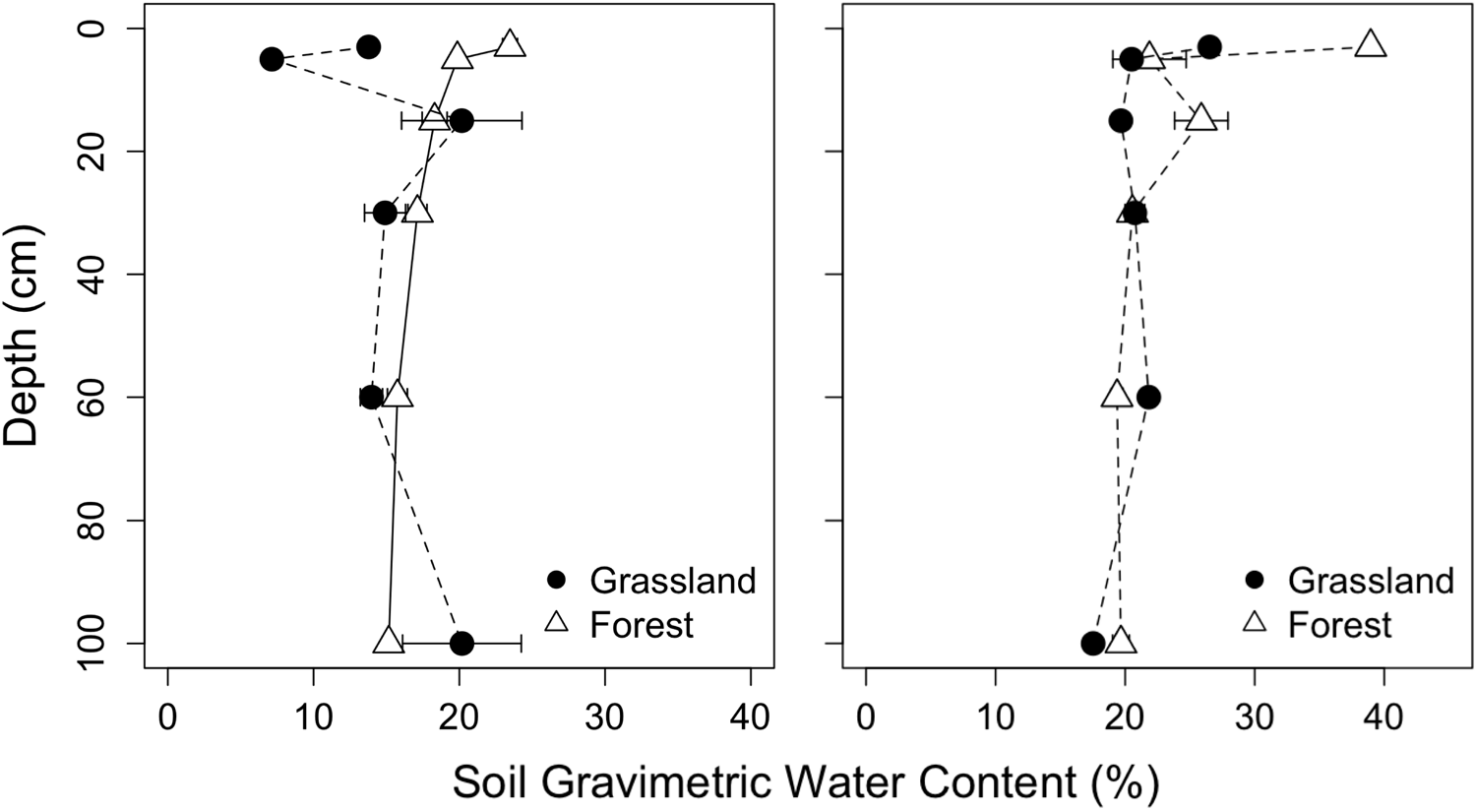
Soil gravimetric water contents (mean ± s.e.) for forest and grassland plots in Barnaul (left) and Tomsk (right).

Soil gravimetric water content at the Barnaul site had a range of 10.6 to 25.1% in the forest and 3.9 to 55.6% in the grassland (Fig. 5). At the Tomsk site, soil water had a range of 14.7 to 39.0% forests and 16.6 to 27.1% in the grassland (Fig. 5). Vegetation and soil depth were not significant in the statistical model while soil moisture content on average was 5% greater at the Tomsk site (p < 0.01).

### Soil δ^13^C profile

**Figure 6:**
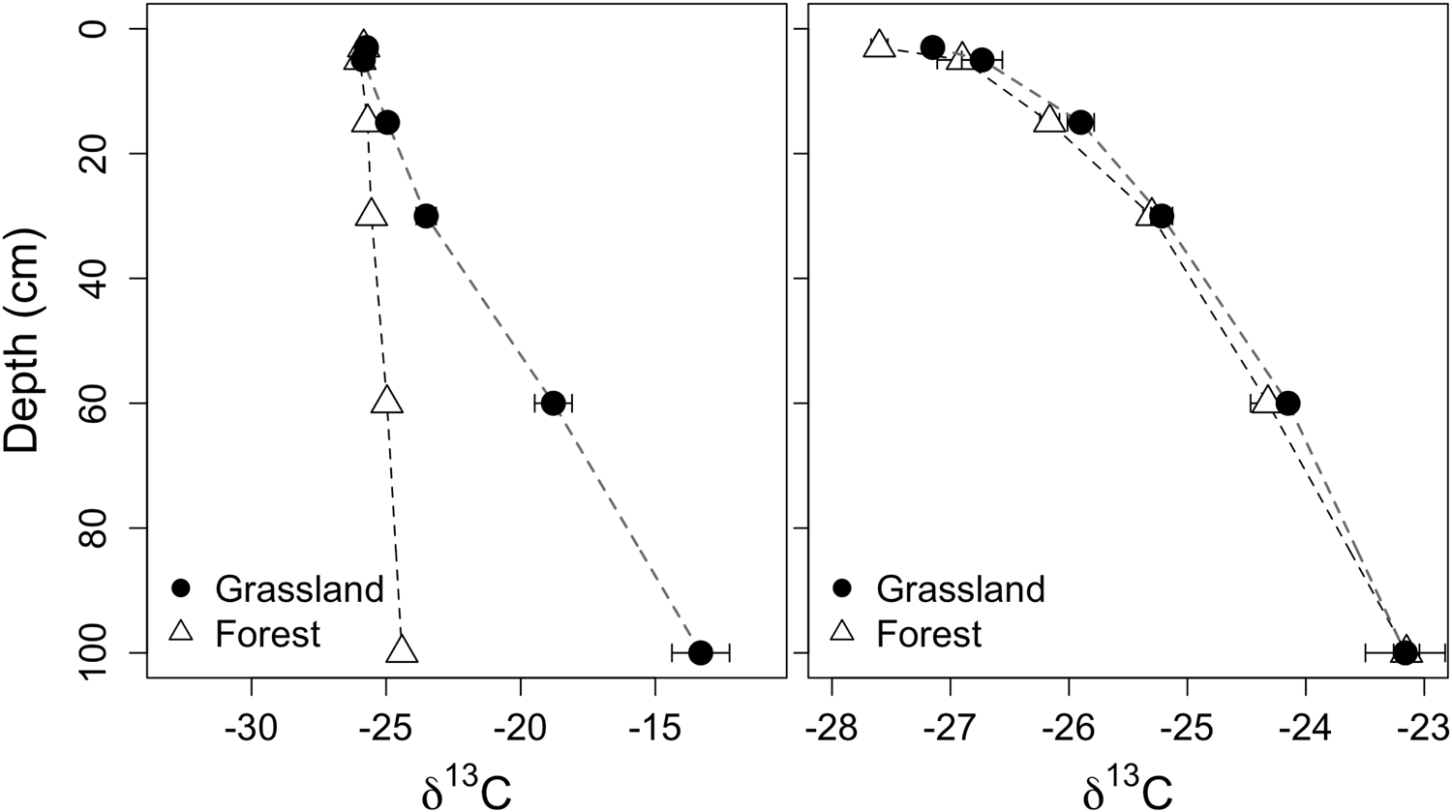
Soil δ^13^C profile of grassland and forest plots at the Barnaul (left) and Tomsk (right) sites (mean ± s.e.).

The patterns of soil δ^13^C were unique to each site and vegetation type (Fig. 6). At Barnaul, the soil organic matter at the surface was more depleted in the heavy ^13^C, which increased with depth. Soil organic matter δ^13^C values in the grassland plots at Barnaul were especially enriched with the heavy isotope approaching values dominated by carbonate. The soil profile patterns at Tomsk were relatively more curve-linear compared to Barnaul. The isotopic values were in general more depleted in the heavy isotope at Tomsk by approximately −2.1‰ (p < 0.01). Differences in vegetation were also significant as grasslands soils tended to be 2.6‰ more enriched in ^13^C (p < 0.01).

### Soil organic matter turnover model

**Figure 7:**
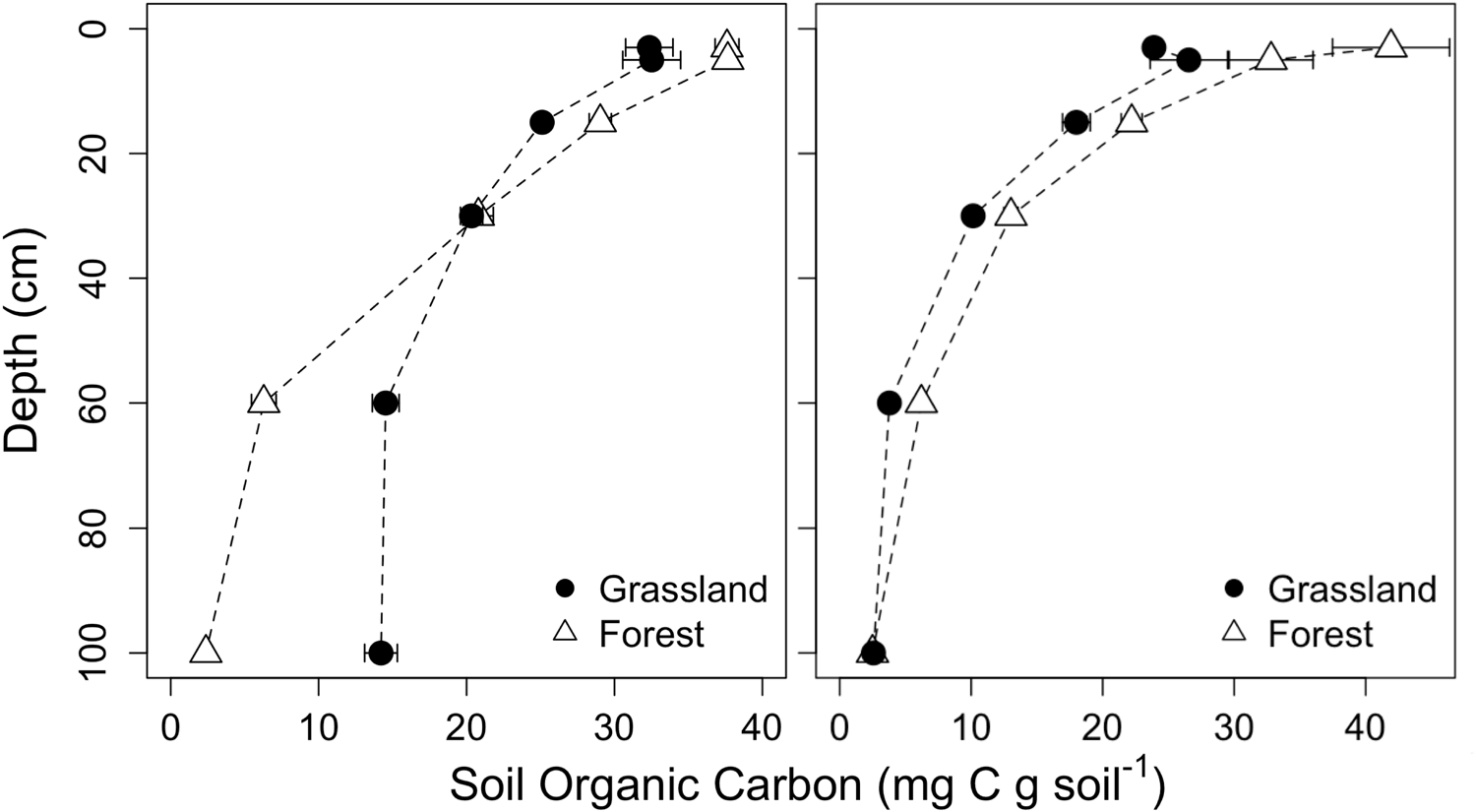
Soil carbon concentration profile of grassland and forest plots at the Barnaul (left) and Tomsk (right) sites (mean ± s.e.).

**Figure 8:**
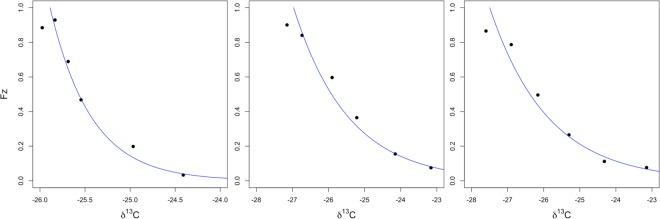
The relationship between relative proportion of carbon present in the soil (*F*_*z*_) and the corresponding δ^13^C (‰) signal: Barnaul forest, Tomsk grassland, Tomsk forest. The relationship was not determined for the grassland at the Barnaul site because of carbonates present within the soil. The turnover parameter (ε) for the different sites are proxies of site biotic and abiotic characteristics.

We fit the soil profile δ^13^C data to a linear model (Table 4). The model could not be applied to the grassland site at Barnaul because of the high degree of secondary carbonates present; however, the remaining vegetation and site data fit both the carbon concentration (r^2^ > 0.91, Fig. 7) and isotope model (r^2^ > 0.96, Fig. 8) well. The difference in *C*_*s*_ between the two sites was not significant, but the difference between the average of the two forests and the grassland site (Tomsk) was (Table 4). When the decomposition term ε is considered, site level differences dominate. There was no detectable difference between the vegetation types at Tomsk, but the difference in ε was significant between the Barnaul and Tomsk forests.

**Table 4:** Soil carbon profile model results.

Parameter	Barnaul	Tomsk	Tomsk
	Forest	Forest	Grassland
Carbon concentration model (eq. 5)			
C_s_ (g C g soil^−1^)	0.041^a*^	0.045^a^	0.029^b^
	0.002^§^	0.003	0.002
Js/D (C cm^−2^ s^−1^)	0.0010^a^	0.0022^b^	0.0011^a,b^
	0.0002	0.0006	0.0003
$\bar{z}$ (cm)	44.10^a^	19.16^a^	27.07^a^
	9.32	4.29	7.45
R^2^	0.91	0.98	0.97
Isotope model (eq. 6)			
D_i_ (‰)	−25.89^a^	−27.50^b^	−29.97^c^
	0.07	0.20	0.16
ε	−0.46^a^	−1.61^b^	−1.52^b^
	0.04	0.13	0.11
R^2^	0.96	0.97	0.97

^*^Estimates followed by the same letter are not significantly different (p < 0.05).

^§^Standard errors are provided below each estimate.

## Discussion

### Soil Water

The overall decrease in δ^18^O values with soil depth is a strong sign of evaporation at all sites and vegetation types. We were able to estimate soil evaporation rates for both vegetation types at the Barnaul site. We report a range in soil evaporation between 0.62 to 0.45 mm d^−1^ for periods during the growing season, which is within the range of other grassland sites with comparative climate in North America^46,47^. This estimate also falls within the range of soil evaporation rates of 0.5 to 1.6 mm day^−1^ measured by lysimeters from this region and elsewhere^18^, although micrometeorological methods estimated larger evaporation rates from boreal forests further north^17,48^. The shallow deviation from the LMWL and the slope of the LEL, support the inference that soil evaporation is not very strong at this site, especially relative to more arid environments^49^ or exposed water bodies^50^. Similar to evaporation, differences in extractable soil moisture were also not significant between the vegetation types indicating sufficient water capacity during the growing season for grassland and tree transpiration. However, our methods aimed to extract as much water as possible, and therefore the water status we report does not necessarily reflect what is available for uptake by plants. At the Barnaul site, properties that control soil water holding capacity will most likely drive future soil water budget response. In this area, the climate is expected to become warmer and drier and economic pressure is increasing to convert grasslands to agriculture. Our results imply that soil water may be available for such changes initially, however, with the potential disruption of soil structure and a shift in water demands to potentially inefficient crop species^51,52^ uncertainty concerning future soil water sustainability remains.

In contrast to the Barnaul site, the Tomsk soil isotopic water profile was unique with respect to the enrichment of water at deeper depths (exhibiting a C-shape profile). This profile prevented robust estimates of grassland evaporation, but we report an estimate of 0.96 mm d^−1^ for the forest plot, which was significantly greater than the rates from forests in Barnaul and refuting our original hypothesis. However, we are cautious with the interpretation due to the reduced profile data we used to arrive at this estimate. Despite this uncertainty, evaporation was clearly present given the enrichment of water in the heavy isotope in the shallow soil surface (3–30 cm). The groundwater in this area is shallow, and groundwater infiltration is the most likely reason that we observed a trend of increasingly enriched soil water isotope values below a soil depth of 40 cm^53^. Another explanation may be that the surface water pool (0–40 cm depth), or the water source that feeds this pool, does not strongly mix with the water pool deeper in the soil, resulting in a deeper water pool with a longer residence time and more enriched isotopic signature^54,55^. Similar to the Barnaul site, soil water at Tomsk did not differ between vegetation types and does not appear to be currently limiting plant productivity. However, the site hydrological dynamics are substantially different between the two sites. The groundwater dynamics observed in this study in tandem with the known surface overflow that occurs at snow-melt^16^ hints at potential drainage challenges if agriculture were to replace the current vegetation and land-use.

The fine root mass density was strongly related to differences in soil water isotopic values, and since soil evaporation rates were not significantly different between site vegetation types, this suggests an important role of plants in explaining these patterns. The δ^18^O of water in grasslands tended to be on the order of 2‰ greater than forests at both sites. The fine root mass density of the forest sites was larger in the deeper profile (>30 cm) than grasslands^30^ (S1) and may facilitate hydraulic redistribution of these deeper and more depleted sources in the profile^56–58^, thus transporting more isotopically depleted water up to shallow soil profile depths. There were also differences at the site level. Interestingly, the Tomsk site tended to have soil water more enriched in the heavy isotopes by about 2‰. Because this site is further north, we would have expected a lower evaporative demand and therefore more *depleted* isotopic values with reference to the southern Barnaul site. However, a higher probability of observing enriched isotopic values at Tomsk is consistent with the C-shaped isotopic profile whereby deeper soil water pools that are enriched in the heavy isotopes may mix with the upper profile by hydraulic lift or with the movement of groundwater.

The overall relationship between the fine root mass density and the soil water isotopic signature highlights the importance of transpiration in the water budget at these sites. While root uptake of soil water does not fractionate water isotopes, the removal of water for transpiration shrinks different water pools belowground, which may exhibit contrasted isotopic signatures. Recent studies have shown that water is held in the soil by different mechanisms (e.g., roots, ion concentrations) resulting in water that is more tightly bound to soil, a non-mobile pool, and a mobile pool which contains water that is more readily available for transport to groundwater or uptake by plants^28,59^. These pools often have distinct isotopic composition in which the mobile pool is better represented by recent precipitation while non-mobile pools are more enriched in the heavy isotopes^28^. We hypothesize that transpiration at our sites removes the mobile pool leaving isotopically heavy water where the fine root mass density is largest; thus, providing a mechanistic basis for the statistical relationship we found in this study.

### Soil Carbon

Similar to the soil water findings, soil carbon differences were primarily driven by site properties. Organic matter turnover, expressed by the term ε in the model^31,36,60^ was not different between the forest and grassland plots at the Tomsk site. Organic matter turnover was about three times faster at the Tomsk grassland and forest plots than the forest site in Barnaul. This finding is consistent with larger leaf litter decomposition rates measured at Tomsk compared to Barnaul^16^. This finding refutes our hypothesis that organic matter turnover would be faster at the Barnaul site given the warmer soil and air temperatures at this southern site. We are cautious in our comparisons between sites however, given the detection of secondary carbonates in the Barnaul grasslands that may also be present in the forest soils. Secondary carbonates, common in loess soils, present in the lower horizons of the Barnaul grassland had a large influence on the soil δ^13^C, but were not as influential within the forest soils. The grasslands are most likely experiencing more leaching than the forest soils, and accumulating secondary carbonates at soil depths greater than 20 cm. Yet, carbonates result in enriched isotopic values deeper in the horizon, increasing the ε term, thus our estimates of organic matter turnover are conservative at the Barnaul site.

The differences in organic matter turnover that we observed may partly result from differences in soil texture. Soil textural properties, such as amounts of clay, silt, and sand, are often associated with organic matter retention and turnover^22,23,61^. For example, slower decomposition is associated with fine soil particles^62,63^, and microbially processed organic material is often present in organo-mineral associations^64^ or by direct interactions with Ca^2+^ or via Ca^2+^ with clay-sized Al-silicates^65,66^. The size fractions between forest and grasslands are not different within a site however, and soil at the Tomsk site contained slightly more silt and metal-oxides than Barnaul^19^ from which we would expect slower decomposition rates. The estimated isotopic values of leaf litter (Di) were significantly different between sites, but the differences between vegetation (4‰) and sites (1.6‰) fall within the natural variability^67^ and do not suggest a shift in vegetation or litter quality. The fact that SOM decomposition is faster at the Tomsk site indicates that other factors, such as site hydrology (including snow levels and leaching) or litter quality, may play a larger role in OM cycling at these sites. Snow levels at the Tomsk site are generally higher than Barnaul^16^, and snow can insulate the soil from freezing^11^. Thus, an additional reason for the relatively faster organic matter turnover at Tomsk is prolonged microbial decomposition of soil organic carbon during periods in winter in which snow prevents the soil from freezing and the subsequent reduction in microbial activity.

### Summary

Water and carbon are intrinsically linked, from ecosystem primary production and respiration to soil nutrient and carbon storage^6,68^. We have shown here that the dynamics of water in soil, including evaporation and shallow groundwater fluctuations, also impact soil organic matter turnover. For our sites in southwest Siberia, we found ample water resources for the present vegetation. The distribution of roots determined, in part, the soil water pools and their stable isotopic composition. At the site spatial scale, soil textural properties that influence leaching and organic matter retention were important drivers of soil carbon turnover. Our study adds to the growing body of work documenting the agricultural potential of this region^19,30,69^ by detailing soil water and carbon processes at our sites. The study serves as a benchmark for future comparisons and the study points to other limitations regarding soil drainage and water table fluctuations that need to be considered when contemplating a shift in land-use toward agricultural use. Lastly, we linked patterns in evaporation and water availability to carbon turnover to provide information that may be useful for nutrient cycling and carbon storage. Future studies are needed that address the influence of crop species or forest conversion on the feedbacks between soil water and organic matter of these forest and grassland ecosystems to help determine the impact of agriculture on the sustainability of plant growth and soil health in this region.

## Electronic supplementary material

Supplementary Information
