# Supplementary material for: Soil evaporation and organic matter turnover in the Sub-Taiga and Forest-Steppe of southwest Siberia
Source: Sci Rep. 2018 Jul 19;8:10904. doi: 10.1038/s41598-018-28977-8 (PMC6053405; doi:10.1038/s41598-018-28977-8)
Supplement: Supplementary file 1 — Supplementary Information [file 41598_2018_28977_MOESM1_ESM.doc]

Supplementary for: Soil evaporation and organic matter turnover in the Sub-Taiga and Forest-Steppe of southwest Siberia

Authors: Zachary E. Kayler, Félix Brédoire, Helene Reickh, Pavel A. Barsukov, Olga Rusalimova, Polina Nikitich, Mark R. Bakker, Bernd Zeller, Sebastien Fontaine, Delphine Derrien

S1. Sample size (*n*) for each profile

| Depth (cm) | Barnaul Grassland | Barnaul  Forest | Tomsk Grassland | Tomsk  Forest |
| --- | --- | --- | --- | --- |
| 3 | 2 | 3 | 3 | 1 |
| 5 | 3 | 2 | 2 | 3 |
| 15 | 5 | 6 | 6 | 4 |
| 30 | 4 | 6 | 6 | 4 |
| 60 | 4 | 5 | 5 | 5 |
| 100 | 4 | 6 | 6 | 5 |

S2. Fine Root Mass Density (mg cm^-3^)

| Barnaul | Forest | Grassland |
| --- | --- | --- |
| 3 | 1.44 | 1.51 |
| 5 | 1.44 | 1.51 |
| 15 | 0.53 | 0.58 |
| 30 | 0.54 | 0.35 |
| 60 | 0.34 | 0.19 |
| 100 | 0.41 | 0.06 |
| Tomsk | Forest | Grassland |
| 3 | 0.89 | 1.06 |
| 5 | 0.89 | 1.06 |
| 15 | 0.58 | 0.21 |
| 30 | 0.23 | 0.04 |
| 60 | 0 | 0 |
| 100 | 0 | 0 |
